# Guy Talk, Precarious Masculinity, and Men’s Sexual Health: A Qualitative Content Analysis of *Men’s Health* Magazine Covers

**DOI:** 10.1177/15579883261418805

**Published:** 2026-01-31

**Authors:** Trenton M. Haltom, Meredith G. F. Worthen

**Affiliations:** 1 Department of Medicine, Baylor College of Medicine, Houston, TX, USA; 2 Department of Sociology, The University of Oklahoma, Norman, OK, USA

**Keywords:** men, magazines, sexual health, precarious masculinity, media

## Abstract

The current study engages precarious masculinity as a framework to examine sexual health in magazines marketed for male audiences. Precarious masculinity is the idea that masculinity is fragile and always being challenged or questioned, particularly considering pressures to defend the social status and privilege associated with masculinity. We conducted a qualitative content analysis of the text or “coverlines” on the covers of *Men’s Health* magazines over nearly 40 years (1986–2024; *N* = 333) of which only 26 (8%) covers address men’s sexual health. Such few coverlines on these topics suggests men’s “visible invisibility,” a concept that describes men’s dominant place in society and the prioritization of their sex lives with women, yet also significant gaps in knowledge, access, and care for men’s sexual health. Our findings reveal three key themes wherein appeals to precarious masculinity are evident: sexual health concerns (e.g., condoms, vasectomies, and prostates), “the truth about testosterone,” and sexual functioning and aesthetics. We identify the verbiage used on the covers of men’s magazines as “guy talk” that involves silly, ironic, or slang-laden verbiage. Using guy talk as a marketing tactic both exposes the precarity of masculinity and serves as a compensatory manhood act that allows men to avoid perceived social consequences associated with expressions of vulnerability and thus preserve masculine privilege. We conclude that the guy talk in men’s magazines that caters to precarious masculinity and, with men’s visible invisibility, does a disservice to men’s health and well-being.

Sexual health and well-being are particularly tricky subjects for men to talk about as topics that bridge anatomy and human development, sexual behaviors, and their consequences (i.e., pregnancy or contracting sexually transmitted infections [STIs]) (Knight et al., 2012; Orenstein, 2020; Waling, 2023; Waling et al., 2023). Given the cultural taboo surrounding sexual health, men turn to “guy talk” that involves silly, ironic, or slang-laden verbiage (Almeling, 2020; Knight et al., 2012; Korobov, 2005, 2009a, 2009b). In the current study, we engage precarious masculinity as a framework to examine sexual health on the covers of issues of *Men’s Health* (United States). Precarious masculinity is the idea that masculinity is fragile and always being challenged or questioned, particularly considering pressures to defend the high social status and privilege associated with masculinity (Connell, 2005; Vandello & Bosson, 2013; Vandello et al., 2008). The result of such precarity engages compensatory manhood acts which help men maneuver to avoid perceived social consequences associated with expressions of vulnerability and thus preserve masculine privilege (Ezzell, 2012; Haltom, 2022; Schrock & Schwalbe, 2009; Sumerau, 2012). Our study identifies “guy talk” on the covers of American *Men’s Health* magazines as a mundane feature of masculinity that enables hierarchical social positioning and reveals the precarity of masculinity (Haltom, 2022; Knight et al., 2012; Korobov, 2005, 2009a, 2009b).

We conducted a qualitative content analysis of the text or “coverlines” on the covers of *Men’s Health* (*n* = 333; 1986–2024) of which only 26 (8%) address men’s sexual health. We suggest that pandering to men with humorous and masculinist colloquialisms may be marketable—and may even contribute some awareness of sexual health—but we can and should do better. As evidence, sexually active men who avoid discussing sexual health have an increased risk of contracting an STI compared with those who do (as well as other health consequences) (Alt, 2002; Courtenay, 2002; Pearson, 2003). Extant research has detailed how masculinities and social contexts influence young men’s sexual health (Gautham et al., 2008; Goldenberg et al., 2008; Orenstein, 2020; Shoveller et al., 2004). Thus, we argue that changing how we both market and talk about men’s health and well-being—especially their sexual health—may be helpful. Put differently, our findings suggest that guy talk in men’s magazines does a disservice to men’s sexual health and speaks to larger issues about how men communicate about their health more generally.

## Literature Review

### Precarious Masculinity

Vandello et al. (2008) describe manhood as precarious, tenuous, and dependent on validation. Because of this, Vandello and colleagues (2008) note how *anything* that challenges one’s manhood can cause anxiety. Indeed, masculinity threats can produce both externalized (e.g., aggression, violence) and internalized responses (e.g., emotional distress, embarrassment, shame, self-harm) (Stanaland et al., 2023). To counteract masculinity threats, men engage in various hypermasculine displays. For example, heterosexual men who experienced challenges to their masculinity are more likely to sexually harass women (Maass et al., 2003) and overestimate their number of past sexual relationships (Cheryan et al., 2015). Heterosexual men also express negativity toward those they perceive as non-masculine (e.g., effeminate men, gay men, or trans people) because even mere association with femininity is threatening to masculinity (Konopka et al., 2021; Pascoe, 2011; Wellman et al., 2021). We generally refer to these as “feminine penalties” given that avoiding femininity is central to the hierarchical organization of masculinity (Connell, 2005). Similarly, Stanaland et al. (2023) explain that those with fragile masculine identities are especially susceptible to feeling threatened and thus respond more intensely when their masculinity comes under question.

### Compensatory Manhood Acts and Men’s Sexual and Reproductive Health

Studies focusing on men, masculinity, and sexual and reproductive health remain scarce (Mohr & Almeling, 2020). The dearth of sexual health discourse has been related to men’s broader lack of investment in their own health and well-being, men’s perceptions that they are “invincible” and thus not susceptible to health issues (especially sexual health problems), and men’s investments in various masculinity tropes (e.g., independence, stoicism, self-reliance) (Connell, 2005; Courtenay, 2000, 2002; Miele & Clarke, 2014; Robertson, 2007). Men also often perceive general, sexual, and reproductive health as the purview of women’s responsibilities (Almeling, 2020; Knight et al., 2012; Littlejohn, 2021).

When men do discuss sexual health, their conversations often center their sexual encounters with women. This form of “guy talk”^1^ helps men—especially those with precarious masculinities—affirm and reify their manhood (Knight et al., 2012). Furthermore, guy talk demonstrates a kind of compensatory manhood act that allows men to maneuver taboo topics or situations in efforts to preserve masculine privilege and legitimacy (Ezzell, 2012; Haltom, 2022; Schrock & Schwalbe, 2009; Sumerau, 2012). For example, teasing humor discourages men to appropriately reflect upon their potentially risky sexual behaviors (e.g., unprotected sex) and their overall sexual health (Knight et al., 2012). Instead of talking about potential sexual problems, men’s sex talk is surface level wherein directly addressing sexual health distracts or dilutes the goal of the conversation: bragging about sexual conquests and pleasure (Knight et al., 2012). While men are open to sharing their sexual behaviors as a way of bolstering their masculine credibility, they are seemingly less interested in discussing their sexual health.

Yet, elements of guy talk that involve humor, irony, and teasing (often at the expense of women) prompts the subject of sexual and reproductive health without challenging masculinity and simultaneously engenders group solidarity (Knight et al., 2012; Korobov, 2005, 2009a). Indeed, men’s serious discussion of sexual health is rare because they feel they must “man up” to engage in such conversations (Knight et al., 2012). Even when men do discuss sexual health with one another, they often use humor to deflect from any potential seriousness or validity of their concerns, thus helping to secure their masculinity and avoid feminine penalties (Knight et al., 2012; Korobov, 2005, 2009a). Thus, for many men, discussions about sexual health are neither possible nor desirable, so they may seek other outlets—like magazines.

### Sexual Health in Men’s Media

Men’s magazines are sites that promote men’s health, well-being, and anxieties, often through the neoliberal discourse that places the onus of health and success on the individual (Barber & Bridges, 2017; Crawshaw, 2007; Dworkin & Wachs, 2009; Lewington et al., 2018). Across magazine studies, common findings emphasize their play on men’s insecurities through self-help articles, advising on ways to become more sexually desirable to women, and exploiting consumerism by placing importance on appearance and grooming (Boni, 2002; Dworkin & Wachs, 2009; Lewington et al., 2018; Ricciardelli et al., 2010; Waling et al., 2018). For example, in their analysis of how four men’s magazines in the United Kingdom discuss penises, Owen and Campbell (2018) suggest that the magazines evoke insecurity and fear in men through over-celebrating penis size and aesthetics thereby highlighting what could go wrong during sex. Such plays on men’s insecurities speak both to how the penis is “masculinity made flesh” and add to the precariousness of masculinity (Owen & Campbell, 2018, p. 333).

In general, media outlets fail to provide information or depictions of sexual health and when they do, they rely on gender stereotypes to bolster men’s sexual appetites and women’s responsibilities for contraception (Hust et al., 2008). When magazines like *Men’s Health* speak to sex, the topics center (hetero)sexual attractions and pleasure-seeking (i.e., orgasms) combined with physical strength (Porter et al., 2017; Waling, 2017). This “her pleasure, his strength” dynamic comes at the cost of sexual or reproductive health. Few studies using magazines for men as data speak to men’s sexual and reproductive health, choosing instead to address other elements of health such as fitness, nutrition, and musculature (Dworkin & Wachs, 2009; for a review, see Waling et al., 2018). In addition, when men’s reproductive health gets communicated to the public, the use of slang (e.g., in describing sperm as “swimmers” and men’s reproductive system as his “junk”) is utilized in a way that presumably appeals to wider audiences (Almeling, 2020). Lacking and low engagement with the subjects of men’s sexual health reveals the precariousness of masculinity and perceived threat to men’s egos despite these outlets’ otherwise wide appeal.

Such poor acknowledgment of men’s sexual health across media outlets aligns with what Waling (2024) describes as “visible invisibility.” Visible invisibility is the idea that, because cisgender heterosexual men maintain a dominant presence in society, their sexual interests have been prioritized across multiple forms of media. Yet, while their sexual interests may prevail, men’s sexual and reproductive health via public health or education initiatives are often missing entirely. Such visible invisibility is negligent to men’s health, those they have sex with, and perpetuates the hegemony of hypermasculine privilege. Thus, it is necessary to investigate the problems associated with media representations of men’s sexual health—or lack thereof.

#### Current Study

The current study examines sexual health by investigating how “guy talk” on the covers of *Men’s Health* may expose the precarity of masculinity and its potential disservice to men’s health. This is especially important because men may turn to outlets like men’s magazines to learn about sexual health independently in efforts to avoid any feminine penalties associated with more public concerns or discussions about sexual health (Knight et al., 2012; Orenstein, 2020; Waling, 2023; Waling et al., 2023). In doing so, we work to expose the significance of men’s magazines as a media outlet that assists men with compensatory manhood acts via guy talk in efforts to appeal to their precarious masculinity in potentially problematic ways.

## Data and Methods

Coverlines (the text on the covers of magazines) are the primary unit of analysis in the current study (Delaney et al., 2016; Grow, 2002; Martin-Biggers et al., 2015). Magazine covers are useful cultural indicators because they are broad representations of magazine content, reflect pertinent cultural moments, and are widely available as units of study (Frederick et al., 2005; Hatton & Trautner, 2011; Jackson et al., 2001; Spiker, 2015). Though covers differ from advertisements, articles, and other content, they still capture the magazine’s essence because they tease, describe, and market the magazine. Put differently, the covers are the most widely viewed element of magazines as they are displayed on newsstands, grocery stores, and published widely online. We conducted a summative qualitative content analysis of the text on the covers of *Men’s Health* as the top magazine in the health and fitness genre (Hsieh & Shannon, 2005; Neuendorf, 2017).^2^

### Data Collection and Sampling

The first issue of *Men’s Health* (United States) was published in 1986, so this was the first year of data collection. We collected issues through 2024 resulting in a census of 38 years of *Men’s Health* (*N* = 333). No publicly available online archive exists for *Men’s Health*.^3^ To circumvent this lack of archive, we used websites like www.coverbrowser.com, a digital gallery of searchable covers assembled from a variety of search engines (e.g., Yahoo and Google) or retailers (e.g., eBay and Amazon). Until recently, *Men’s Health* has consistently been issued about 10 times a year,^4^ which allowed us to determine any missing issues in the data set.

*Men’s Health* commonly produced newsstand and subscriber covers or released multiple covers of the same issue for wider reach or for collectors (Johnson, 2002). We determined which images to use based on the quality of the image (e.g., some images were obstructed due to mailing labels). When necessary, we used e-merchant websites (e.g., eBay, Amazon) to collect the clearest images of the covers and to supplement any missing months. We prioritized newsstand versions of the covers because they serve to bring in new readers by telling them more of what is inside the magazine and often contain more text (Husni, 2009). When the magazines issued multiple covers in the same month, we analyzed them all; while the text rarely differed across these versions, the cover model did. Coverlines tease or summarize the full text articles which often have different titles. The full text of magazines is rarely publicly available without purchase. Instead, databases like Gale or EBSCO provide short descriptions through university libraries. We describe the content of the article affiliated with the coverline when available with access provided from an academic institution when licensing allows. The first author collected data from each cover (for more details, see Haltom, 2021). We focus only on *Men’s Health* issues with sexual health cover lines here.

### Coding and Analyses

The initial coding categories were developed from a literature review of magazines and advertisements that used content analytic methodologies (Goffman, 1976; Hatton & Trautner, 2011; Waling et al., 2018). We coded for both manifest content which is more obvious and straightforward and latent or subtle and subjective content (Chapman, 1983; Neuendorf, 2017, p. 31; Potter & Levine-Donnerstein, 1999). Our approach is what Potter and Levine-Donnerstein (1999) call “projective content” because this coding relied on coder interpretations and knowledge of the content in the cover lines. Both authors iteratively contributed to the sorting and thematic organization of coverlines reported in results.

## Results

Out of 333 *Men’s Health* covers across 38 years (1986–2024), only 26 (8%) address men’s sexual health. Among these coverlines, we find three key themes: sexual health concerns, “the truth about testosterone,” and sexual functioning and aesthetics. We provide the coverlines from these magazines and their issue dates in Table 1.

**Table 1.** Descriptions of *Men’s Health* Covers (1986–2024) with Sexual or Reproductive Health Coverlines (*N* = 26).

	*n*	%
*Covers with One or More Models* (*n* = 26)		
1	23	88
2+	3	12
*Total Cover Models*	29	—
*Gender*^a^ (*n* = 28)		
Men	27	96
Women	1	4
*Model Gender and Race/Ethnicity* (*n* = 28)^a^		
White Men	21	75
White Women	1	4
Men of Color	6	21
Women of Color	0	0
*Cover Models* (*n* = 29)		
Celebrities	12	41
Unnamed Models	17	59
*Image Framing by Cover* (*n* = 26)		
Headshot (above the beltline)	6	22
Full Body (below the beltline)	20	77
*Image Style by Cover* (*n* = 26)		
In-Action	17	65
Posed	9	35

a An infant appeared on one cover. We do not include their gender here because it is undeterminable based on the image.

### Describing the Content on the Covers

With magazines, it is impossible to take the text and image out of context. In Table 2, we provide descriptive statistics regarding the images across the sample of covers containing sexual health coverlines. While we focus on the coverline text in our main analyses, there are observable patterns related to precarious masculinity in the images to consider.

**Table 2.** Sexual and Reproductive Health Coverlines in *Men’s Health* (1986–2024) Organized Thematically (*N* = 26).

Year	Month	Model	Cover Line
**Sexual Health Concerns** (*n* = 11)			
1986	Winter	Unknown	The Great Condom Test of 1986
1987	February	Unknown	How to Beat a Herpes Attack
1988	Winter	Randy Travis	Vasectomy change-of-heart insurance
1990	April	Unknown	Plus Dustin Hoffman, mountain bikes, hair care, new condoms
1994	July-August	Unknown	The great condom test
1997	February	Unknown	Protect your prostate
1998	November	Unknown	Save your own privates
2001	July	Unknown	Your prostate protection plan
2001	December	Unknown	Birth control The no-condom solution
2017	May	Unknown	Protect Your Prostate
2022	March	Joseph Baena	Health Check 2.0 Online Docs Meds in the Mail A Bot for Your Prostate
“**The Truth about Testosterone**” (*n* = 9)			
1994	November	Unknown	The truth about testosterone
2000	December	Unknown	Testosterone Top off your tank—naturally
2007	September	Jamie Foxx	What’s sapping our testosterone? Don’t let the global decline strike at your house!
2010	December	Mark Wahlberg	Testosterone power boost! Tap your secret source
2012	December	Jamie Foxx	Electric Testosterone!
2012	September	Liam Hemsworth	24-hour testosterone boosters
2013	March	Adam Levine	Boost metabolism! Build muscle! Spike testosterone!
2019	October	Arnold Schwarzenegger & Gabriel Luna	Muscle! Sex! Energy! The New Rules of Testosterone
2022	January-February	Jacob Elordi	Testosterone Nation Do you really need more T in your life?
**Sexual Functioning & Aesthetics** (*n* = 6)			
1993	May	Unknown	Sex Rx Love lessons on video
1996	June	Unknown	New male sex drug
1998	July-August	Unknown	Did circumcision ruin your sex life?
2001	November	Unknown	Exclusive! Sex and your city Lusty or limp?
2019	May	Terry Brown	Sex + CBD
2019	June	Joe Manganiello	Special Report You and Your Penis

*Note*: We attempt to preserve the original punctuation and capitalization of the coverlines throughout.

Not surprisingly, the vast majority of models across *Men’s Health* covers referencing sexual health are men (*n* = 27, 96%) and when a woman appears, it is only once (4%) and she is depicted negatively: she is whispering into a man’s ear with her fingers crossed as he smiles next to the coverline “how women lie to men.” Men on the covers are mostly white (*n* = 21, 75%). Men of color appear on a fifth of covers (*n* = 6, 21%). Cover models were most often unnamed (*n* = 16; 57%), the rest were celebrities (*n* = 12, 43%). On all but three covers (12%), a single person appears. In addition, all of the cover images depict men’s bodies as capable and muscular, a sharp shift from fit, unnamed men in action (e.g., on bikes, climbing, or swimming) to primarily stationary and posed muscular shirtless men in the late 1990s and with more celebrities in the 2000s (Haltom, 2021). Overall, the main patterns across these cover images demonstrate value in individuality, whiteness, and muscularity leaving little space for other representations of masculinity. In doing so, these cover images appeal to men’s precarious masculinity by displaying a narrow albeit stereotypical image of “healthy” men’s bodies allowing men to avoid any potential risks of moving beyond their prototypical comfort zone and thus preserve masculine privilege (Ezzell, 2012; Haltom, 2022; Schrock & Schwalbe, 2009; Sumerau, 2012).

### Thematic Findings

#### Sexual Health Concerns

Our first theme, sexual health concerns, includes mentions of condoms, vasectomies, prostate health, and STIs (*n* = 11).

Condoms were mentioned on only four covers of *Men’s Health*. “The Great Condom Test” comes up twice—once in the first issue of *Men’s Health* in 1986 and again in the July-August 1994 issue. The full article from 1986 is unavailable, although the coverline appears ironically next to an image of a nude father and his similarly naked infant (Daniels, 2013). In 1994, the associated article by Mark Roman (1994) was a grading and ranking of nine condoms according to their features, durability, and usage. There are also mentions of “new condoms” (April 1990), the full title of the article references a “Condom update. . .: from the land of small cars come the big prophylactic and other options” (full text unavailable) (Pechter, 1990). The article associated with the coverline “Birth control The no-condom solution” (December 2001) is actually about vasectomy as an option for male contraception, and not condoms at all but rather the lack of need for condoms (Stump et al., 2001). Calling attention to condoms in these infrequent though light-hearted ways emphasizes men’s resistance to condoms and low uptake (Davis et al., 2014).

Some coverlines use guy talk to address sexual health. Alliteration about the prostate was common, for example. Several times coverlines encourage readers to “protect your prostate” (February 1997; May 2017) or create “your prostate protection plan” (July 2001). Associated articles addressed prostate-specific antigen testing to detect prostate cancer and new research on the relationship between testosterone and prostate cancer (Gaul, 2001; Martin, 1997; Stewart, 2017). Though catchy, such alliteration yields a jovial vibe, suggesting that either prostate health should not be taken seriously—or that this is the only way to communicate to men about prostate health.

As another way of diminishing the significance of sexual health while also alluding to STIs, *Men’s Health* uses militaristic language to masculinize the topic because the military and masculinity are so analogous. “Save your own privates” appears on *Men’s Health* in November 1998 and serves as a double *entendre* for men’s genitals with the Academy Award-winning film *Saving Private Ryan* also released in 1998. Using similarly militaristic language, a coverline from February 1987 provides guidance on “How to Beat a Herpes Attack” (February 1987), as if herpes is a military attack on the body rather than an outbreak of blisters and lesions from the herpes simplex virus. The common thread across these coverlines are the use of masculine tropes and deflection of sexual health.

#### “The Truth about Testosterone”

Our second theme addresses references to testosterone on *Men’s Health* covers (*n* = 9), a subject first mentioned on the November 1994 issue that claims to uncover “The Truth about Testosterone.” Interestingly, in the early 2000s, a shift occurred in sexual health-related coverlines from those about condom use to testosterone. This is perhaps related to the production of injectable testosterone and gel application alongside global increases in testing and prescriptions of testosterone in the early 2000s (Handelsman, 2013; Nieschlag & Nieschlag, 2019; Walsh et al., 2015).

Over the years, *Men’s Health* has relied on masculinist language particularly in reference to testosterone. Use of alliterative language like “Testosterone Top off your tank—naturally” (December 2000) and using phrases like “electric testosterone!” (December 2012), “24-hour testosterone boosters” (September 2012), the “testosterone power boost” (December 2010) or how to “spike testosterone” (March 2013) all emphasize male performance and its athletic potential. Similarly, a cover from January-February 2022 featuring actor Jacob Elordi calls upon “Testosterone Nation,” begging the question, “Do you really need more T in your life?” Testosterone Nation is a “community for enhanced fitness” that boasts multiple social media channels and over 442,00 community members on its forum and website (T Nation, 2025). Such language engrains the importance of testosterone to masculinity and fuels men’s insecurities about testosterone levels all the while obscuring the purpose of assessing testosterone.

#### Sexual Functioning & Aesthetics

Our third theme refers to sexual functioning via erections and penile aesthetics across six covers. *Men’s Health* coverlines use humor to speak to these subjects while also reflecting the importance men place on these symbols of masculinity and virility (Owen & Campbell, 2018). For example, to lighten the otherwise uncomfortable subjects of circumcision and impotence, a July-August 1998 cover begs the question “Did circumcision ruin your sex life?” In the associated article cheekily entitled, “Separated at Birth,” journalist Mark Jenkins (1998) outlines debates about infant circumcision, myths about the relationship between circumcision and masturbation, and health rationale for circumcision. A November 2001 article proposes the question “Lusty? or Limp?” referring to erections in their “Exclusive” on “Sex and your city.” The article is about “the best [and worst] cities for men” in an editorial ranking the “healthiest—and deadliest—places to be a guy” with categories related to health (e.g., heart disease, obesity, and cardiovascular fitness), sex (e.g., sexually transmitted diseases and erectile dysfunction), traffic (e.g., car accidents and commuter times), and many others (Marion, 2001, p. 110). In the erectile dysfunction category, the “stiffest” city is Phoenix while the “Hardest Place to Stay Hard” is Miami (followed by Baltimore, Washington, D.C., New Orleans, and Tulsa), a rating determined by the ratio of men to urologists and treatment rates for erectile dysfunction.

A May 1993 issue also uses the silly “Sex Rx” rhyme to prompt “Love lessons on video,” too. These “love lessons on video” refer to reviews various adult sex-education videotapes (Steinberg, 1993). In a more direct reference to sexual aids, an “ask her anything” column with Naomi Piercey (2019) who speaks to incorporating cannabidiol (CBD) into one’s sex life. For the first and only time, a cover directly mentions the penis without silly obfuscation through a “Special Report” about “You and Your Penis” on a cover from June 2019 featuring actor Joe Manganiello. This June 2019 issue is full of articles about men and their penises including “The State of the American Penis” (Dukoff, 2019), “What Your Penis is Telling You Right Now” (E. S., 2019), “Big Dick Problems” (McCammon, 2019), “The Penis Diet” (Roussell, 2019), among others. Given the vulnerable nature of these subjects, these rhetorical strategies and the brevity of the coverlines may help initiate these conversations while simultaneously appealing to men’s insecurities.

## Discussion

Our analysis of *Men’s Health* coverlines revealed evidence of “guy talk” (i.e., using silly, alliterative, and masculinist language) about men’s sexual health across three themes: sexual health concerns, “the truth about testosterone,” and sexual functioning and aesthetics. We argue that these tactics are indicative of efforts to address the precariousness of masculinity by focusing on humor and obfuscation to help men avoid any feminine penalties that may be associated with exposing their own sexual health vulnerabilities (Knight et al., 2012; Korobov, 2005, 2009a). As a marketing ploy to draw in readers or subscribers, this strategy is a compensatory manhood act that justifies men’s maneuvering around difficult subjects or scenarios in ways that bolster masculine privilege (Ezzell, 2012; Haltom, 2022; Schrock & Schwalbe, 2009; Sumerau, 2012).

That only 8% of *Men’s Health* covers over nearly 40 years is troubling and reveal how men’s magazines do a disservice to sexual health information, consistent with other recent magazine studies that have evaluated the efficacy of their fitness and health recommendations (Jalloh et al., 2020). In particular, men’s magazines relegate sexual health to a side issue unworthy of serious focus bolstered by the paucity of sexual health content in these magazines. Our findings reflect the precariousness of masculinity as sexual and reproductive health topics appear to threaten men and masculinity or else they would be mentioned more frequently and openly in this space reserved for men’s interests.

The infrequency of sexual and reproductive health coverlines appeals to Waling’s (2024) concept of “visible invisibility.” That is, while media outlets generally showcase heterosexual men making their manhood and masculinity highly visible, such visibility and the social privilege associated with it allow for the obfuscation of men’s sexual health. This visible invisibility dynamic supports our findings and the precarity of masculinity such that critiques of men’s health, virility, or sexual functioning cause such anxiety that they are nearly avoided altogether by outlets like magazines that purport to report on men’s health (Connell, 2005; Miele & Clarke, 2014; Vandello & Bosson, 2013; Vandello et al., 2008).

With masculine precarity in play, we can begin to understand why guy talk could be an effective way of communicating. By addressing sexual health using guy talk, men’s magazines appeal to those who may feel insecure about their sexual health problems. Humor may help translate complicated medical terminology that often comes alongside health advice (Newman, 2005), while also simulating discourse among friends (Benwell, 2001). Humor in magazines for men is also not new as Benwell’s (2001) analysis of “laddish” banter in magazines published in the 1990s in the United Kingdom reveals. Yet, in the case of the magazines studied here, humor is perhaps an equalizer that takes the edge off the taboo subject of sexual health. Conveying that sexual health is inconsequential through guy talk may help assuage internalized anxieties (i.e., masculinity threats) associated their poor sexual functioning and performance, for example. In these ways, men’s magazines may be especially likely to pander to the precariousness of masculinities while also offering ways of addressing otherwise taboo topics.

Nevertheless, guy talk may do a disservice to men. Encouraging men to talk explicitly about their sexual health may help formulate healthy masculinities—both medically in terms of their health and socially in terms of encouraging vulnerability among men about stigmatized subjects. Scholars who have explored the concept of healthy masculinities suggest that men employ them out of a need to bolster other valued elements of masculinity and thus discount femininity in ways that promote gender inequalities (O’Brien et al., 2005; Waling, 2019). For example, when men do seek health care, it is out of preservation and restoration of other valued elements of masculinity like sexual functioning (O’Brien et al., 2005). While perhaps true when men see a doctor privately, addressing the subjects of sexual health head-on in various public media forms remain invisible (Waling, 2024).

It is also interesting to assess how magazines speak to sexual health—if at all. It makes sense that *Men’s Health* might address men’s sexual health given its focus though the infrequency in which they do so is troubling. Readers of *Men’s Health* are presumably men who are more engaged in their health, bodies, and well-being and thus more attuned to these subjects regardless of their taboo. Notably, no cover of *Men’s Health* referred to the HIV/AIDS despite its start at the height of the epidemic in the 1980s, the consequence of which erases the cultural significance of HIV/AIDS and the population most affected by it (i.e., gay men) (an irony not lost on these authors given the number of shirtless, muscular men who appear on the covers). Such omission is indicative of the pervasive cultural narrative of HIV/AIDS as a “gay disease” and that the presumed audience of these magazines are heterosexual men whose masculinity need not be further threatened by suggestions that they read about gay men’s diseases. This is precarious (hetero)masculinity and visible invisibility at work.

### Implications

Our findings speak to important media implications. First, because our findings show that most representations of sexual health in popular men’s magazines pander to men using silly and non-specific guy talk to mask and even deter serious conversations about sexual health, we suggest men’s magazines and larger media conversations catering to men should move away from this format. Instead of appealing to humorous references and slang terms about penises, medically specific language that emphasizes the significance of men’s health concerns is needed in men’s magazines and coverlines. If men are to understand the importance of sexual health, we need to speak about it *as if it is important*. We argue that frank, open discussions about men’s sexual health and well-being are possible—and that men may even appreciate such an approach because it highlights their needs as significant and valuable while moving away from adolescent banter. Waling (2024) offers a similar recommendation in talking to men about sexual accountability such that broaching the topics of sexual and reproductive health using “care and understanding of their lived experiences . . . invites them into dialogues” (p. 260) (particularly given evidence that “talking at them” is ineffective). This “growing up” of men’s media may even contribute to greater sexual health awareness which is important because sexually active men who avoid serious discussions of sexual health have an increased risk negative sexual health consequences (Alt, 2002; Courtenay, 2002; Pearson, 2003).

Second, images of masculinity should be expanded to include more diverse representations of men’s health. EXPAND

Third, more broadly, men-focused media content—whether online or in print—should rethink their approach to communicating to men and masculinity in general. Our study identifies precarious masculinity-informed guy talk on the covers of men’s American magazines as enabling a culture of misinformation and ignorance about men’s sexual health. However, beyond this, feelings of precarious masculinity can result in a variety of other compensatory manhood acts (Ezzell, 2012; Schrock & Schwalbe, 2009; Sumerau, 2012), the goal of which aid maneuvers to avoid perceived social consequences associated with expressions of vulnerability and thus preserve masculine privilege (Haltom, 2022). Building out a more inclusive or expansive discussion of masculinity that encourages men to explore the diversity of manhood, embrace the spectrum of masculinities, and engage in a wider conversation than what is traditionally represented men’s media could have a more global impact on improving not only men’s sexual health, but men’s social and mental health as well.

### Limitations

There are limitations to this study that are worth noting. First, only one magazine was examined in the current study. Incorporating additional magazines in a similar genre into future explorations would be beneficial, however, the scope of the dataset across four decades is significant and unique among magazine studies that too often analyze only a few years (for a review, see Waling et al., 2018). Moreover, our reporting of the paucity of sexual health coverlines from a magazine that touts men’s health should be viewed similarly as reporting negative or non-significant results from quantitative or statistics-based studies—with context, negative results can say a lot about a phenomenon. Second, we do not know how men respond to these coverlines; additional qualitative work interviewing men’s responses to magazine coverlines would be especially informative (e.g., Delaney et al., 2016; Martin-Biggers et al., 2015). Third, though beyond the scope of the current study, a deeper investigation into the magazine’s article content would be interesting. Examining how the coverlines may be mimicked or challenged in the associated articles could expose the relationship between the covers and articles. Fourth, while some may critique our use of magazine covers as data, they remain significant marketing tools whether in print on newsstands or in digital formats online (Pershan, 2021). Even in the digital age as physical newsstands decline, former editor of *New York* magazine, Adam Moss believes covers remain the “brand statements,” the “voice” of the publication, and can be distributed widely on social media as advertisement for the magazine (Husni, 2018).

## Conclusion

Overall, our analysis of the coverlines on *Men’s Health* magazines exposes the clear use of guy talk across all three of our themes: sexual health concerns, “the truth about testosterone,” and sexual functioning and aesthetics. In doing so, we identify this marketing tactic as both a successful appeal to men’s precarious masculinity as well as a form of justification for maneuvering around difficult topics in ways that bolster masculine privilege without exposing potential weaknesses (Ezzell, 2012; Haltom, 2022; Schrock & Schwalbe, 2009; Sumerau, 2012). Ultimately, we conclude that using guy talk to discuss men’s sexual health is damaging to men’s well-being because it obscures the significance of these important subjects. To conclude: “guy talk” should be “real talk.”

## Notes

1. While the topics may overlap, “guy talk” differs from “locker room talk” as the latter prioritizes the sexual denigration, objectification, harassment of, and violence against women that occurs in homosocial settings like men’s locker rooms (Curry, 1991; Leone & Parrott, 2019; Simeone & Jeglic, 2019).

2. Data come from a larger mixed-methods study of GQ, Men’s Health, and Sports Illustrated (*N* = 2750) (Haltom, 2021)

3. There is evidence to suggest an archive once existed and has been since been removed from online perhaps due to criticisms that *Men’s Health* editor David Zinczenko (2000–2012) repeatedly used the same cover lines and heavy reliance on the same cover template over several years (Cook, 2009; Quigley, 2009).

4. *Men’s Health* typically publishes a winter issue for January-February and a summer issue for July-August resulting in 10 issues a year.
